# A106 COMPARING SIZE MEASUREMENTS OF SIMULATED COLORECTAL POLYPS SIZE AND MORPHOLOGY GROUPS WHEN USING A VIRTUAL SCALE ENDOSCOPE OR VISUAL SIZE ESTIMATION: A BLINDED RANDOMIZED CONTROLLED TRIAL

**DOI:** 10.1093/jcag/gwac036.106

**Published:** 2023-03-07

**Authors:** C Haumesser, M Zarandi-Nowroozi, M Taghiakbari, R Djinbachian, M Abou Khalil, S Sidani, J Liu, B Panzini, I Popescu Crainic, D von Renteln

**Affiliations:** 1 Montreal University Hospital Research Center; 2 University of Montreal Medical School; 3 Division of Internal Medicine, Montreal University Hospital Center (CHUM); 4 Division of Gastroenterology, Montreal University Hospital Center (CHUM), Montreal, Canada

## Abstract

**Background:**

The polypectomy technique used to remove colorectal polyps is influenced by the size of the polyp. Furthermore, the criteria for assigning surveillance intervals after polypectomy are based on size and pathology results. Visual size assessment is potentially fraught with being inaccurate. The virtual scale endoscope (VSE) allows projection of a virtual scale onto colorectal polyps allowing real-time size measurements.

**Purpose:**

We studied the relative accuracy of VSE compared to visual assessment (VA) for the measuring simulated polyps of different size and morphology groups.

**Method:**

We conducted a blinded randomized controlled trial using simulated polyps imbedded within a colon model. Sixty simulated polyps were created and evenly distributed across four different size groups (0-4.9 mm, 5-9.9 mm, 10-19.9 mm and ≥ 20 mm) and 3 different Paris morphology groups (flat, sessile and pedunculated). Six endoscopists (3 staff gastroenterologists and 3 trainees) performed size measurements of all sixty simulated polyps using random allocation of either VA or VSE.

**Result(s):**

A total of 359 measurements were completed. The relative accuracy of VSE was significantly higher when compared to VA for polyps ≥ 5 and <10mm, ≥ 10 and <20mm, ≥ 20mm (p=0.004; p<0.001, p<0.001). For polyps <5mm, the relative accuracy of VSE compared to VA was nominally higher (79.4% versus 74.1%) but this was not statistically significant (p = 0.186). The relative accuracy of VSE was higher when compared to visual assessment for sessile (p = 0.001), flat (p < 0.001) and pedunculated polyps (p = 0.002). VSE misclassified a lower percentage of ≥ 5 mm polyps as < 5 mm (2.9%), ≥ 10 mm polyps as < 10 mm (5.5%) and ≥ 20 mm polyps as < 20 mm (21.7%) compared to visual estimation (11.2; 24.7 and 52.3% respectively; p=0.008, p<0.001 and p=0.003).

**Conclusion(s):**

VSE had significantly higher relative accuracies for every polyp size group or morphology type aside from diminutive where VSE had a non-significantly higher relative accuracy. VSE enables endoscopist to better classify polyps into correct size categories at clinically relevant size thresholds of 5-, 10- and 20-mm. Implementing VSE as a standard measurement tool could allow improving clinical decision making for accurate surveillance interval assignment and choice of polypectomy technique.

**Please acknowledge all funding agencies by checking the applicable boxes below:**

Other

**Please indicate your source of funding;:**

The study was supported by a "Fonds de Recherche du Québec Santé" career development award (Daniel von Renteln) and a University of Montreal student award “PRogramme d’Excellence en Médecine pour l’Initiation En Recherche – PREMIER (Claire Haumesser).

**Disclosure of Interest:**

C. Haumesser: None Declared, M. Zarandi-Nowroozi: None Declared, M. Taghiakbari: None Declared, R. Djinbachian: None Declared, M. Abou Khalil: None Declared, S. Sidani: None Declared, J. Liu: None Declared, B. Panzini: None Declared, I. Popescu Crainic: None Declared, D. von Renteln Grant / Research support from: ERBE Elektromedizin GmbH, Ventage, Pendopharm, Fuji and Pentax, Consultant of: Boston Scientific Inc., ERBE Elektromedizin GmbH, and Pendopharm

